# Choroidal metastasis: Impact of primary tumors and age on survival - a single center analysis

**DOI:** 10.1371/journal.pone.0354598

**Published:** 2026-07-27

**Authors:** Philipp Fuchs, Adrian Reumueller, Judith Kreminger, Elena Zafeiri, Stefan Sacu, Reinhard Told, Roman Dunavoelgyi

**Affiliations:** Department of Ophthalmology and Optometry, Medical University of Vienna, Vienna, Austria; Fondazione Policlinico Universitario Agostino Gemelli IRCCS, ITALY

## Abstract

**Purpose:**

The purpose of this study was to determine whether patient’s age and their primary tumor type act as independent predictors of their survival after a diagnosis of choroidal metastasis.

**Methods:**

This retrospective single-center study (August 2013 – August 2025) included 70 patients with choroidal metastases. Clinical data and multimodal imaging were extracted from medical records. Tumor volume was calculated from ultrasound measurements. Patients were grouped according to primary tumor origin (lung, breast, or other primaries). Survival was compared between these three primary tumor groups and according to age. Group differences in continuous variables were assessed using ANOVA/Kruskal-Wallis testing, and survival distributions were analyzed with Kaplan-Meier curves. Patients were categorized into younger (<59.6 years) and older (≥59.6 years) groups for Kaplan-Meier survival analysis. Univariate and multivariate Cox regression models were performed to identify independent predictors of overall survival.

**Results:**

Median overall survival was 71.6 weeks. Statistically significantly longer survival was found in patients <59.6 years (median 88.3 weeks; estimated 3-year survival 33.2%) compared with those ≥59.6 years (median 41.9 weeks; 3-year survival 16.8%; p = 0.037. Primary tumor group analysis showed a median survival of 94.4 weeks for breast cancer (estimated 3-year survival 38.3%), 52.7 weeks for lung cancer (9,5%), and 40.6 weeks for other primaries (19.7%). However, this difference was not statistically significant (p = 0.269). In multivariate Cox regression analysis, age ≥ 59.6 years (HR 2.10; p = 0.016) and >1 extraocular metastases elsewhere in the body (HR 2.53; p = 0.029) were independent predictors of mortality.

**Conclusion:**

Survival in choroidal metastases is strongly driven by age and the number of extraocular metastatic sites at presentation. Our findings suggest that younger age and a lower metastatic burden are most reliable indicators for a more favorable prognosis.

## Introduction

Although metastases are common in both lung and breast cancer, ocular involvement is rare. In breast cancer it occurs in 0.5% of cases and represents 2–3% of metastatic presentations, most often choroidal or orbital [1]. Lung cancer is also a major source of intraocular metastases, again predominantly to the choroid, but absolute incidence remains low relative to the total burden of metastatic disease [2,3]. It has been reported that 1–7% of patients with lung cancer develop intraocular metastasis [4,5].

Despite being relatively rare, intraocular metastases represent the most prevalent type of intraocular malignancy in adults [6,7]. An estimated 1–2% of all cancer patients develop intraocular metastases, with higher rates detected histologically than clinically [8]. The choroid represents the primary site of metastatic involvement (90%), followed by iris (8%) and ciliary body (2%) [6]. The highly vascular and low-flow characteristics make the uveal tract susceptible to metastatic tumor cell deposition [9,10]. In rare cases, manifestations may also appear in the retina, optic disc, vitreous and lens capsule [11–15]. Uveal metastasis predominantly originate from primary cancer of the breast (37–47%), lung (21–50%), with other less common origins including kidney, gastrointestinal tract, cutaneous melanoma, lung carcinoid, prostate, thyroid, pancreas and other sites [6,16–18]. The overall prognosis is unfavorable, with a 5-year survival rate of 24%. Patients with pancreatic metastases have the poorest survival (mean, 4.2 months), whereas those with lung carcinoid metastases demonstrate the most favorable prognosis (92% at 5 years) [19]. Median survival after diagnosis of choroidal metastasis is 10–12 months for breast and lung cancer [6].

This study aims to provide a comprehensive overview of choroidal metastases of a single center and to report characteristics in relation to primary tumor type and patient age at choroidal metastasis diagnosis, and further to evaluate survival outcomes.

## Methods

This retrospective study was conducted at the Department of Ophthalmology and Optometry, Medical University of Vienna. Data were collected retrospectively from medical records and imaging databases of consecutive patients diagnosed with choroidal metastasis between August 2013 and July 2025. The study was approved by the Institutional Review Board by the Medical University of Vienna (Vienna, Austria) Ethics Committee (EK 1401/2022), which waived the requirement for informed consent due to the retrospective nature of the study. Medical records were accessed for research purposes between December 01, 2025, and January 31, 2026. During the initial data collection phase, the authors (P.F. and E.Z.) had access to information that could identify individual participants. However, all data were pseudonymized prior to statistical analysis, and no identifying information was included in the final dataset or used in this report to ensure data security and participant anonymity. The study was conducted in adherence with the principles of the Declaration of Helsinki and Good Clinical Practice (GCP) guidelines.

Inclusion criteria were clinical and/or imaging-based diagnosis of choroidal metastasis, patients with known or unknown primary malignancy, availability of medical records and follow-up period within the timeframe August 2013 – October 2025. Patients were excluded if they had primary uveal melanoma, iris or ciliary body metastasis, or no follow-up information available. Patients who had metastasis in both eyes were included with data from right eyes only. Examinations were conducted by experienced ocular oncologists using slit-lamp biomicroscopy and multimodal imaging at each visit. The diagnosis of choroidal metastasis was established based on characteristic clinical and imaging features. Imaging modalities included wide-field fundus photography, ultrasonography, fundus autofluorescence, and optical coherence tomography (OCT), complemented by fluorescein, indocyanine green, and OCT angiography when clinically required. Demographic information included age (years) and sex (male/female). Collected data comprised the clinical features of choroidal metastasis, the applied treatment (methods), and information regarding the primary tumor, including its treatment before the diagnosis of metastasis and time of death. Death dates (and, where available, ICD-10 causes of death) were obtained via the Sterbedatenabgleich service (Death data linkage) of the Medical University of Vienna, which matches patient records with the official death data from Statistik Austria [20]. Clinical features included tumor dimensions and the presence of retinal fluid or retinal detachment in slit lamp examination.

Data describing tumor dimensions were extracted from B-scan ultrasonography. Ultrasonographic measurements included one transverse still frame through the apex of the lesion and one longitudinal still frame encompassing the greatest tumor extent. Imaging was performed using a 20-MHz probe for intraocular assessment. Measurements were obtained with the system’s caliper function. Tumor diameters on transverse and longitudinal scans were defined as straight lines between opposing tumor borders, while tumor thickness was measured from the apical tumor surface to the inner scleral boundary. Tumor dimensions were recorded as thickness (mm), width (mm, transverse/tangential), and length (mm, longitudinal). Tumor volume (mm³) was calculated using the ellipsoidal model formula: (π/6 × length × width × height) [21].

### Statistics

All groups were tested before statistical analysis for normal distribution using Kolmogorov Smirnoff test. If data was normally distributed, a one-way ANOVA was performed; otherwise, the Kruskal-Wallis test was used. Statistical analyses were performed using SPSS (Version 29.0. Armonk, NY: IBM Corp., USA).

Choroidal metastases originating from 14 subgroups of primary tumors were analyzed. Overall survival was calculated from date of first diagnosis of choroidal metastasis until death. Survival distributions were generated using the Kaplan-Meier method and group differences in survival were assessed with the log rank test. Patients who were alive at the time of the last follow-up or were lost to follow-up were right-censored at the date of their last clinical examination. Comparisons were made by primary tumor type and age. For Kaplan-Meier and Cox regression analyses, primary tumors were categorized into three groups to ensure adequate sample sizes and reliable survival estimates:

Group 1 included all patients with primary lung cancer (metastatic non-small-cell and small-cell carcinoma). Group 2 included primary breast cancer (all metastatic mammary carcinomas). Group 3 included all other primary tumors gastrointestinal (colorectal and pancreatic), genitourinary (renal, prostate, bladder and gynecologic; each being metastatic), and head and neck/endocrine/melanoma primaries.

### Cox Regression Analysis

To identify independent predictive factors associated with overall survival, a multivariate Cox proportional hazards regression model was performed. Continuous variables included age at the time of choroidal metastasis diagnosis, tumor volume and height, while categorical variables included number of other non-ocular metastasis elsewhere in the body (≤1 vs. > 1), tumor classification (lung, breast, or others), presence of subretinal fluid/retinal detachment (yes/no), systemic therapy prior to metastasis (yes/no), and order of diagnosis (eye first vs. primary tumor first). Univariate Cox regressions were conducted first to screen for potential predictors. In univariate Cox regression analyses, age at metastasis was analyzed as a continuous variable to assess the general effect of increasing age on survival. For multivariate modeling, age was dichotomized at the sample median (59.6 years) to facilitate clinical interpretation and maintain balanced group sizes. Variables with a significance level of p < 0.15 in univariate analyses or with strong clinical relevance were entered into the multivariate Cox model. Sex was excluded from the multivariate analysis due to its complete overlap with tumor classification (all breast cancer patients were female), which would otherwise introduce collinearity. The number of non-ocular metastases elsewhere in the body was tested in univariate analysis. Despite the small subgroup size (n = 13, eyes with 0 or 1 non-ocular metastasis elsewhere in the body), the variable was retained in the multivariate model due to its biological relevance as a marker of disease burden. Consequently, results should be interpreted with caution. Results were expressed as hazard ratios (HR) with 95% confidence intervals (CI), and a p-value less than 0.05 was considered statistically significant.

## Results

This study included a total number of seventy patients and seventy eyes, with ten patients presenting choroidal metastases in both eyes. Baseline primary tumor type classification of the patient cohort are presented in Table 1, while demographic characteristics are summarized in Table 2. The clinical characteristics of patients with choroidal metastasis are described in Table 3. There were statistically significant differences in mean age at diagnosis of metastasis (p = 0.021) and systemic therapy prior to choroidal metastasis diagnosis (p = 0.013). Age at diagnosis of choroidal metastasis was statistically significantly lower in patients diagnosed with breast cancer as compared to lung cancer (mean difference = 8.77 years, p = 0.024), this difference did not remain statistically significant for multiple comparisons using the Bonferroni correction (adjusted α = 0.017). Systemic therapy prior to metastatic presentation was significantly more common in breast cancer than in lung cancer (p = 0.005) (Bonferroni-adjusted α = 0.013).

**Table 1:** Classification of primary tumors according to organ systems.

Characteristics	Overall (n = 70)	N
1. Lung cancer	Non-small-cell and small-cell carcinoma	28
2. Breast cancer	Invasive breast carcinoma (ductal and lobular types)	23
3. Other tumors	Colorectal carcinoma (3), pancreatic carcinoma (1), renal cell carcinoma (3), bladder carcinoma (1), prostate carcinoma (3), gynecologic carcinomas (2), laryngeal carcinoma (1), oral cavity carcinoma (1), parotid gland carcinoma (1), thymic carcinoma (1), mixed glandular-thymic carcinoma (1), cutaneous melanoma (1)	19

**Table 2:** Demographic characteristics of patients with choroidal metastasis. Values are presented as mean ± standard deviation (SD) and median with interquartile range (IQR). The asterisk (*) indicates statistical significance at p < 0.05*.

	Lung Cancer (n = 28, 40%)	Breast cancer (n = 23, 32.9%)	Other tumors (n = 19, 27.1%)	Total (n = 70, 100%)	P
Mean Age (±SD), age at metastasis diagnosis years, (median, IQR)	64.6 (±10.71), (64.25, 15.5)	55.83 (±11.34), (56.83, 18)	57.54 (±13.02), (59.58, 15.33)	59.8 (±12.09), (60.17, 17.42)	0.021*
Female sex, n (%)	15 (53.6%)	23 (100%)	5 (26.3%)	43 (61.4%)	–
Bilateral ocular manifestation, n (%)	6 (21,4%)	2 (8.7%)	2 (10.5%)	10 (14.3%)	0.373
Unileratal ocular manifestation, n (%)	22 (78.6%)	21 (91.3%)	17 (89.5%)	60 (85.7%)	0.373
Multiple choroidal metastases per eye, n (%)	3 (10.7%)	6 (26.1%)	0 (0%)	9 (12.9%)	0.039
Choroidal metastasis detected before primary tumor diagnosis, n (%)	8 (28.6%)	1 (4.3%)	3 (15.8%)	12 (17.1%)	0.072
Number of non-ocular metastasis elsewhere in the body (>1), n (%)	21 (75%)	21 (91.3%)	15 (78.9%)	57 (81.4%)	0.313
Systemic therapy prior to choroidal metastasis diagnosis, n (%)	11 (39.3%)	18 (78.3%)	13 (68.4%)	42 (60%)	0.013*
Symptomatic at presentation, n (%)	14 (50%)	14 (60.9%)	13 (68.4%)	41 (58.6%)	0.437

**Table 3:** Clinical characteristics of patients with choroidal metastasis. Values are presented as mean ± standard deviation (SD) and median with interquartile range (IQR). The asterisk (*) indicates statistical significance at p < 0.05*.

	Lung Cancer	Breast cancer	Other tumors	Total	P
Width mm, mean (±SD), Median (IQR)	11.42 (±4.72), 11.6 (6.89)	12.13 (±4.64), 11.61 (8.21)	12.84 (±4.46), 11.7 (6.85)	12.08 (±4.46), 11.6 (6.07)	0.613
Length mm, mean (±SD), Median (IQR)	10.88 (±4.78), 9.9 (6.25)	11.27 (±4.89), 11.29 (6.27)	11.8 (±3.65), 11.9 (5.4)	11.29 (±4.44), 11.15 (5.62)	0.813
Height mm, mean (±SD), Median (IQR)	4.19 (±3.04), 3.3 (1.99)	2.99. (±2.28), 2.61 (1.49)	4.49 (±2.12), 3.75 (2.84)	3.87 (±2.59), 3.26 (2)	0.007*
Volume mm^3^, mean (±SD), Median (IQR)	460.74 (±689.99), 234.03 (368.61)	342.59 (±574.89), 178.48 (275.79)	442.1 (±390.71), 280 (620.81)	418.29 (±567.61), 223.59 (341.75)	0.794
SRF visible	26 (92.9%)	11 (47.8%)	16 (84.2%)	53 (75.7%)	<0.001*

For the analysis of ultrasound tumor dimensions, measurements were available of 59 patients for width, 58 for length, 62 for height, and 58 for volume. The analysis of tumor dimensions revealed no significant differences regarding width (p = 0.613, ANOVA) or length (p = 0.813) between the three primary tumor groups. Tumor height differed statistically significantly between groups (Kruskal-Wallis test, p = 0.007), whereas no significant difference was observed for tumor volume (p = 0. 794). Post-hoc pairwise comparisons (Mann-Whitney U test) showed statistically significant differences in height between the lung cancer and the breast cancer group (p = 0.038), and between group 2 (breast cancer) and group 3 (other tumors) (p = 0.002). However, after Bonferroni correction for multiple testing (adjusted α = 0.017), the only statistically significant difference remaining was between breast and the “other” group; whereas the difference between breast and lung cancer group lost statistical significance. There was a statistically significant difference in SRF visible in the three subgroups (p < 0.001). Pairwise comparisons were adjusted using the Bonferroni correction (α = 0.017). SRF was significantly more frequent in lung cancer compared with breast cancer (p < 0.001) and in the “other” tumor group compared with breast cancer (p = 0.014), whereas lung cancer and the “other” group did not differ significantly (p = 0.345).

### Kaplan Meier Survival depending on Primary Tumor Types

At the time of analysis, 48 of 70 patients (68.6%) had deceased. Event occurrence of death by primary site was 19/28 (67.9%) in lung cancer, 14/23 (60.9%) in breast cancer, and 15/19 (78.9%) in other tumors. The mean survival time was 115.3 weeks for lung cancer, 205.7 weeks for breast cancer, and 112.3 weeks for other primary tumors. Median survival was 52.7, 94.4, and 40.6 weeks. For the whole study group, the mean survival time was 158.2 weeks, and the median was 71.6 weeks. The mean observation period from the diagnosis of choroidal metastasis until death or last follow-up was mean 92.9 weeks (SD 120.18 weeks).

**Fig 1:**
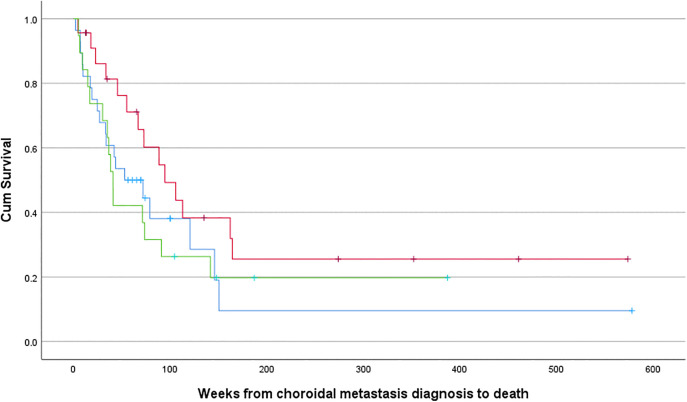
Kaplan Meier survival curves according to primary tumor group: 1 (blue) lung cancer, 2 (red) breast cancer, 3 (green) other tumors.

There was no statistically significant difference in overall survival between the three groups (p = 0.269). The estimated 1-year survival rate was 53.6% in lung cancer, 76.2% in breast cancer and 42.1% in the other primary tumor group. At 3-years, the estimated survival rate was approximately 9.5% for the lung cancer group, 38.3% for the breast cancer group, and 19.7% for the other tumor group. At 5-years, the estimated survival rate was approximately 9.5% for the lung cancer group, 25.5% for the breast cancer group, and 19.7% for the other tumor group. The estimated overall 1-year, 3-years and 5-years survival rates for the whole study cohort were approximately 56%, 24% and 18%. The Kaplan Meier survival curve can be seen in Figure 1.

### Kaplan Meier Survival in Age

When dichotomizing age at the group median (59.6 years), 32 patients were classified as younger and 38 as older than the median. At the time of analysis, 18/32 younger patients (56.3%) and 30/38 older patients (78.9%) had experienced the event (death). The mean survival time was 209.5 weeks (median 88.3 weeks) for patients younger than 59.6 years, and 118.7 weeks (median 41.9 weeks) for those aged 59.6 years or older. The estimated 1-year, 3-years and 5-years survival was approximately 70.5%, 33.2% and 26.5% in patients younger than 59.6 years and 46.2%, 16.8% and 12.6% in patients aged ≥59.6 years.

**Fig 2:**
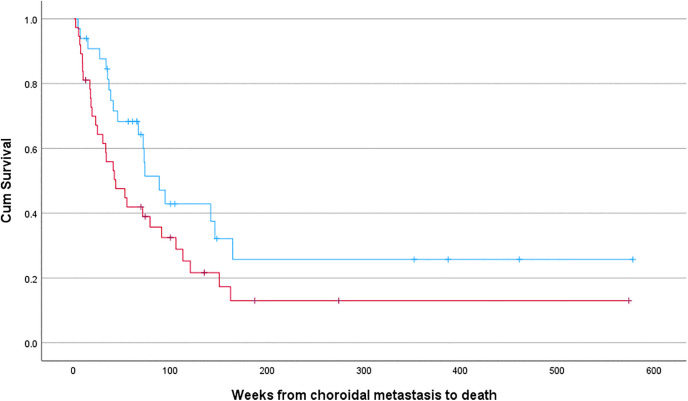
Kaplan Meier survival curves according to age group (median split at 59.6 years), blue: younger (<59.6years) red: older (≥59.6 years).

According to the Log-Rank test, the difference in survival between the two age groups was statistically significant (p = 0.037), indicating a longer survival in the younger group. The Kaplan Meier survival curve can be seen in Figure 2.

### Univariate Cox regression analysis

Univariate Cox regression analyses were conducted to identify variables associated with overall survival. Variables were selected based on clinical relevance and prior evidence from the literature. A trend toward reduced survival was observed with increasing age at metastasis (HR = 1.025; 95% CI 0.999–1.051; p = 0.056).

Patients with more than one non-ocular metastasis elsewhere in the body had an approximately 1.9-fold higher risk of death compared to those with a single or no metastasis elsewhere in the body (HR = 1.87; 95% CI 0.83–4.18; p = 0.129).

Both variables were therefore included in the multivariate model. Tumor classification (lung, breast, or others) was not significantly associated with survival (*p* = 0.276) but was included due to biological plausibility.

Sex was not a significant predictor (HR = 1.53; 95% CI 0.86–2.74; p = 0.148) and was excluded from the multivariate model due to collinearity with tumor type (all breast cancer patients were female). Other clinical and ocular factors including systemic therapy before metastasis, subretinal fluid or serous retinal detachment, tumor volume, tumor height, and timing of primary cancer diagnosis (choroidal metastasis vs. primary tumor) showed no significant association with survival and were not included for further analysis.

### Multivariate Cox Regression Model

A multivariate Cox proportional hazards model was performed including primary tumor group (lung, breast, other), age at metastasis (2 groups: median split 59.6 years) and number of other non-ocular metastasis elsewhere in the body (≤1 vs. > 1).

The multivariate Cox proportional hazards regression model was statistically significant (p = 0.013), indicating that the included variables collectively improved the prediction of survival. Among the individual predictors, both age group (median split at 59.6 years) and number of non-ocular metastases elsewhere in the body were statistically significant. Patients aged ≥59.6 years had a 2.1-fold higher hazard of death compared with those younger than 59.6 years (HR = 2.10; 95% CI: 1.15–3.82; p = 0.016). Likewise, patients with more than one metastasis elsewhere in the body had a statistically significantly increased risk for mortality (HR = 2.53; 95% CI: 1.10–5.83; p = 0.029). The primary tumor classification (lung, breast, others) did only show a non-significant trend but did not reach statistical significance (p = 0.088).

## Discussion

This retrospective analysis of seventy patients diagnosed with choroidal metastasis focused on the influence of ‘primary tumor type’ and ‘age at the time of choroidal metastasis diagnosis’ on overall survival. Our findings confirm that younger age (<59.6 years) is a significant independent predictor of prolonged survival, whereas older age is associated with a significantly higher hazard of death. In contrast, the primary tumor type did not reach strict statistical significance as a predictor of overall survival, but demonstrated a notable trend in our multivariate analysis.

Studies showed that prognosis following ocular metastasis is poor, with median survival typically <12 months. While local ophthalmic treatments achieve high rates of intraocular tumor control, the systemic prognosis remains consistently poor due to the underlying disseminated disease [1,2]. Consequently, factors beyond ocular management become more clinically relevant – in particular the effect of patient age and the biological characteristics of the primary tumor, both of which may significantly influence survival once ocular metastatic spread has occurred.

### Effect of age on patient survival

In our findings, age at choroidal metastasis diagnosis was lower in patients diagnosed with breast cancer by a mean of 8.77 years compared to lung cancer (p = 0.024). These results are consistent with previous reports by Shields et. al [6,22].

Our observation that younger age is associated with prolonged survival is strongly supported by d’Abbadie et al., who identified age ≥ 50 years as a significant predictor of treatment failure and linked mortality risk directly to metastatic involvement in the liver or pleura/lungs [23]. Consistent with this, our data showed that patients with more than one metastasis elsewhere in the body had a statistically significantly increased risk for mortality. Interestingly in our data, we could show that younger adults (<59.6) had significantly longer survival rates which is in contrast to Shields et al [22]. Multiple factors may explain this discrepancy. First, Shields et al. utilized clinically established and predefined age categories, which are ideal for characterizing such an extensive population. Whereas we applied a data-driven cutoff, specifically optimized for our cohort. We observed survival trends that may reflect the specific characteristics of our patient population, complementing the broader findings reported in larger series. Second, their study spans 1974–2017, when systemic cancer therapies evolved considerably; younger patients in our more recent cohort likely benefited from modern treatments. Furthermore, younger patients typically present with fewer comorbidities, better functional status, and less extensive metastatic disease, which allows more intensive systemic therapeutic strategies [22–24]. Third, Shields et al [22]. reported substantial differences in primary tumor distributions across age groups (e.g., higher rates of lung cancer in older adults), but did not adjust for this in multivariate models. In our analysis, older age remained an independent predictor of mortality after adjustment for metastatic burden and primary tumor.

Together, these methodological and clinical differences may account for the contrasting age-related survival patterns between the two studies.

### Effect of primary tumor type on patient survival

In the literature, breast cancer (37–47%) and lung cancer (21–50%) are reported as the most common primary sites [6,16–19]. Our findings align with previously published data, confirming that the majority of choroidal metastasis arises from breast (32.9%) and lung (40%) malignancies.

Survival after diagnosis of choroidal metastasis is poor and varies depending on the primary tumor type. In the literature the overall survival rates of patients with different primary tumors differ between publications. Our observed 3-year survival rates (9.5% in the lung cancer group, 38.3% in the breast cancer group, and 19.7% in the “other tumors” group) support the trend that lung cancer metastases are associated with a lower survival compared to breast cancer metastasis which is supported by the literature [5,19,25–27]. While univariate analysis in Kaplan Meier survival curve did not reach statistical significance for primary tumor types (Kaplan Meier p = 0.269), the multivariable Cox model (p = 0.013) – which adjusts for age, non-ocular metastasis elsewhere in the body, and primary tumor type – showed a non-statistically significant trend (p = 0.088). We interpret this cautiously, as our sample size and number of events within each primary tumor subgroup were limited, and the study is likely underpowered to detect modest differences in survival.

One important limitation of this study is the relatively small sample size of 70 patients. While the primary tumor origins were stratified into three main groups—breast, lung, and others—less common primary sites were combined into a heterogeneous ‘other’ category. This ‘other primary tumors’ category includes a wide spectrum of different tumor origins (12 different rare malignancies) that exhibit varying metastatic behaviors and survival patterns [19]. Due to the rarity of these entities, which represents a consecutive cohort from the largest tertiary referral center in Austria, separate statistical analysis for each rare tumor type was not feasible. While grouping them into a single category is a limitation regarding specific prognostic forecasting, omitting these patients would have introduced a severe selection bias and impaired the real-world representation of our data. Grouping patients as ‘Others’, as it has been published in this context previously by Shields et al [19,22] a feasible way to address rare real-world data in ocular oncology. Furthermore, the overall limited sample size and the subsequent formation of even smaller subgroups for categorical variables (e.g., n = 13 for patients with ≤1 non-ocular metastasis) mathematically resulted in wide confidence intervals in our multivariate analysis; therefore, these specific hazard ratios should be interpreted with appropriate clinical caution. However, it is worth noting that the analysis was conducted retrospectively over a period from 2013 to 2025 in one specialized center, which may lead to a more homogeneous and targeted patient selection. Another limitation was the retrospective character of this study, which might introduce potential selection and information bias. Moreover, exact volumetric ultrasound data could not be included in the analysis, as historical clinical documentation primarily focused on tumor height rather than full three-dimensional measurements.

Although the presence of multiple extra-ocular metastases was a significant predictor of mortality, their specific anatomical locations were not separately analyzed to prevent statistical fragmentation and loss of power. Furthermore, our study is limited by the lack of molecular and histological subtyping of the primary malignancies (hormone-receptor or HER2 status in breast cancer, and specific oncogenic mutations in lung cancer). Given the retrospective design and a long inclusion period beginning in 2013, alongside the fact that many patients were referred from external institutions across different federal states, centralized access to primary tumor pathology reports or tissue samples was unavailable. While we acknowledge that molecular subtypes are critical confounders for overall systemic survival, our analysis focuses on easily accessible clinical and ophthalmic parameters that can guide the ocular oncologist at the time of presentation.

## Conclusion

In conclusion, this study expands the current knowledge on prognostic factors in choroidal metastases. Younger patients showed significantly longer survival, whereas the primary tumor type did only show a non-significant trend but did not reach the level of significance. Age and systemic metastases (>1) were independent predictors of survival. Breast cancer had the longest median survival, lung cancer the shortest. Existing published literature on age and primary tumor subgroup effects in choroidal metastasis is still limited, highlighting the relevance of these findings.
