# Supplementary material for: Choroidal metastasis: Impact of primary tumors and age on survival - a single center analysis
Source: PLoS One. 2026 Jul 27;21(7):e0354598. doi: 10.1371/journal.pone.0354598 (PMC13405101; doi:10.1371/journal.pone.0354598)
Supplement: Attachment — Submitted filename: Response to Reviewers.docx [file pone.0354598.s002.docx]

| **Suggestion, Question,**  **or Comment from the**  **Editor** | **Author’s Response** | **Change in the Manuscript** |
| --- | --- | --- |
| Dear Authors, please revise your manuscript according to comments provided by the Reviewers, particularly addressing issues regarding the heterogeneity in group three and better depicting primary tumor histology | We thank the Editor for managing our manuscript and for highlighting these critical areas. We have carefully revised the manuscript according to all comments provided by the reviewers. A detailed, point-by-point response to every reviewer comment is provided below.  Regarding the specific issues raised: We have expanded our Limitations section to better adress the lack of detailed primary tumor histology and molecular subtyping, which is a typical limitationt of long-term, multi-regional retrospective cohorts.  **Regarding the heterogeneity in Group 3** ("Other"), we completely understand the concern. We respectfully opted to retain this group in the current revision because omitting these patients entirely would result in the loss of valuable, real-world data regarding rare choroidal metastases. Grouping rare entities in this manner is a methodologically accepted approach in the ophthalmic oncology literature (Shields 2018: MidEastAfrJr) to preserve the integrity of the entire study population without misrepresenting the true clinical diversity of these patients. **However, we are entirely open to omitting Group 3 from the analysis if the editorial team and reviewes ultimately concern it strictly necessary.** |  |
| **Suggestion, Question,**  **or Comment from**  **Reviewer #1** | **Author’s Response** | **Change in the Manuscript** |
| The manuscript is very well written and a good point to make. Would accept as is, adds to the information already known for metastases and helps discuss prognosis with patients that come in with questions. | We sincerely thank the reviewer for this highly positive feedback and the recommendation for acceptance. We are very pleased that our primary objective - providing a practical tool to assist clinicians in prognostic patient counselling - resonated so well with you. Your encouraging words are deeply appreciated. |  |
| **Suggestion, Question,**  **or Comment from**  **Reviewer #2** | **Author’s Response** | **Change in the Manuscript** |
| The uvea is the most common site of metastasis in the eye. For both ophthalmologist and general practitioners, this article provides highly valuable information that predicts the survival of patients with uveal mets resulting from primary tumours of the breast or lung and also relates the age of the patients at the time of uveal mets to the survival. The clinical implications of this paper are that we can better counsel our patients about their prognosis. | We thank the reviewer for this highly encouraging summary and for recognizing the clinical objective of our work. It was our goal to translate retrospective survival data into a practical, evidence-based framework for everyday clinical practice.  By identifying clinical parameters - such as patient age at the time of choroidal metastasis diagnosis and the primary tumor entity - we hope to empower ophthalmologists, oncologists, and general practitioners. Providing these disciplines with reliable prognostic tools is essential to offer tailored, realistic, and empathetic counseling to patients navigating this challenging stage of their disease. We are grateful that the clinical value and practical implications of our study resonated so well with you. |  |
| **Suggestion, Question,**  **or Comment from**  **Reviewer #3** | **Author’s Response** | **Change in the Manuscript** |
| Fuchs and colleagues present results of a retrospective analysis of survival in patients with metastatic tumors to the choroid (CM). They found that older patient age and more than one choroidal lesion were predictors of mortality. The study is interesting in its concept as a counseling basis for patients with choroidal metastasis.  However, several integral methodological flaws limits the ability to draw any solid conclusions. | We sincerely thank the reviewer for his/her time, important evaluation, and constructive feedback on our manuscript. We are pleased that the reviewer highlights the clinical value of our study as an interesting and much-needed "counseling basis" for patients suffering from choroidal metastases. Providing clinician-guided prognostic framework at the time of ophthalmic presentation is indeed the primary objective of our work.  We completely acknowledge that a retrospective study design over a long inclusion period carries methodological challenges and limitations, particularly regarding the availability of historical molecular tumor subtyping and varying follow-up durations across different regions.  However, we would like to emphasize that our study reflects a consecutive, real-world cohort from the largest tertiary ophthalmic oncology referral center in Austria, Europe. To address the valuable methodological points raised, we have revised the manuscript. Specifically, we have:   - Clarified how the Kaplan-Meier method mathematically handles varying follow-up durations via right-censoring to eliminate survival data distortion. - Expanded the Limitations section to transparently discuss the necessary heterogeneity of the "Other" primary tumor group and the lack of molecular subtyping, noting that these are common limitations of long-term retrospective multi-regional data in rare diseases. - Refocused the discussion to highlight that this study isolates easily accessible clinical and ophthalmic parameters to serve as an immediate, practical counseling tool for the ocular oncologist. - Added symptomatology   Below, we provide a point-by-point response to each specific methodological concern and outline the corresponding changes made to the revised manuscript. |  |
| **Major Comments** |  |  |
| The major concern in the study is the lack of subtyping of the primary tumors, which is a major confounder for survival. For example, breast cancer is not a single disease but a heterogenous group of diseases. Triple negative disease has poor survival than ER/PR tumors or HER-2 enriched. Similarly, lung cancer has many subtypes with different survival patterns. The lack of incorporation of molecular subtypes introduces great bias in the results. | We entirely agree with the reviewer that molecular and histological subtyping (such as receptor status in breast cancer or oncogenic drivers in lung cancer) severely impacts systemic prognosis and represents a known confounding factor in survival analyses.  However, due to the retrospective nature of our study and the inclusion period spanning back to 2013, complete molecular subtyping was unfortunately not feasible or available. As the primary tertiary referral center for ocular oncology in Austria, a substantial portion of our patients are referred from external hospitals and different federal states. Consequently, we lack direct access to external comprehensive oncological electronic health records or historical biopsy tissue blocks required to systematically reevaluate tumor subtypes from over a decade ago.  To acknowledge this important point, we have explicitly added this concern to the limitations section. We now emphasize that while our study provides a robust overview of clinical and ocular predictors, the lack of molecular stratification is a limitation to long-term, multi-regional retrospective cohorts in rare diseases. | **Added to the manuscript Page 9 Line 39 - Page 10 Line 5:** Furthermore, our study is limited by the lack of molecular and histological subtyping of the primary malignancies (hormone-receptor or HER2 status in breast cancer, and specific oncogenic mutations in lung cancer). Given the retrospective design and a long inclusion period beginning in 2013, alongside the fact that many patients were referred from external institutions across different federal states, centralized access to primary tumor pathology reports or tissue samples was unavailable. While we acknowledge that molecular subtypes are critical confounders for overall systemic survival, our analysis focuses on easily accessible clinical and ophthalmic parameters that can guide the ocular oncologist at the time of presentation. |
| The other major confounder not accounted for is the sites of extra-ocular metastasis; for example, metastatic disease to the brain carries much worse prognosis than to the bones. | We thank the reviewer for raising this highly relevant clinical point. We completely agree that the specific anatomical location of extra-ocular metastases plays an important role in systemic survival.  However, given our overall cohort size and the high variability of extra-ocular metastatic sites among these patients, incorporating specific anatomical locations into the current multivariate survival model was not statistically feasible. Doing so would have resulted in severe statistical fragmentation and a loss of power, preventing us from drawing reliable conclusions alongside the other primary variables.  Because we recognize the immense prognostic importance of this specific confounder, we are currently planning a dedicated follow-up study where the distribution and distinct prognostic impact of specific extra-ocular metastatic sites will be the primary research question. We have acknowledged the lack of site-specific analysis of extra-ocular metastases as a limitation in the revised manuscript. | **Added in the manuscript Page 9 Line 37-39:** Although the presence of multiple extra-ocular metastases was a significant predictor of mortality, their specific anatomical locations were not separately analyzed to prevent statistical fragmentation and loss of power |
| As the authors acknowledge in the limitations, group 3 is heterogenous ("Other"); each of the 12 malignancy types would carry different prognosis and grouping all types under one category is inappropriate. This third group should have been omitted. | We thank the reviewer for raising this important point regarding the heterogeneity of the "Other" group. We have already mentioned this in our limitations. While we completely agree that this group comprises various tumor entities with distinct systemic prognoses, we respectfully argue that omitting these patients entirely would introduce a significant selection bias and misrepresent the clinical reality of choroidal metastases.  Choroidal metastasis is a rare condition. As the largest tertiary ophthalmic oncology center in Austria, our cohort of 70 patients collected over a long-term period reflects the true epidemiological distribution. In clinical practice, rare primaries (such as kidney, bladder or thyroid carcinomas) do present to ocular oncologists, and excluding them would artificially homogenize the cohort, reducing the real-world utility of our survival data.  Statistical power limits our ability to analyze all 12 sub-entities individually. Grouping them as "Other" is a standard and methodologically accepted approach in ophthalmic oncology literature for managing rare sample sizes while preserving the entire study population (ocular metastasis). To address the reviewer’s valid concern, we have expanded our Limitations section to explicitly emphasize that the "Other" category serves as a collective baseline for non-mammary and non-pulmonary entities, and its results should be interpreted as a reflection of overall clinical diversity rather than a single disease entity. This approach has also been used previously by Shields et at. grouping rare disease as others (Shields 2018: MidEastAfrJr). **If required by the reviewers and editor we of course can omit the group “other”.** | **Added to the Manuscript Page 9 Line 20-26:** Due to the rarity of these entities, which represents a consecutive cohort from the largest tertiary referral center in Austria, separate statistical analysis for each rare tumor type was not feasible. While grouping them into a single category is a limitation regarding specific prognostic forecasting, omitting these patients would have introduced a severe selection bias and impaired the real-world representation of our data. Grouping patients as ‘Others’, as it has been published in this context previously by Shields et al is a feasible way to address rare real-world data in ocular oncology. |
| There was no minimum limit on follow-up duration mentioned by the authors. In that sense, patients with recent CM diagnosis were included and compared to patients with long term diagnosis, which would skew survival data. | We appreciate the reviewer's comment, but we respectfully disagree that including patients with shorter follow-up times skews the survival data.  One of the fundamental mathematical advantages of the Kaplan-Meier method is its ability to handle varying follow-up durations through right-censoring. Patients with a recent diagnosis who are still alive do not distort the long-term survival probabilities; they contribute to the "number at risk" for the exact duration they are observed and are appropriately censored at their last follow-up.  Introducing an arbitrary minimum follow-up limit would cause severe selection bias (specifically immortal time bias or left-truncation bias), as it would artificially exclude patients who pass away shortly after diagnosis. To provide an unbiased, real-world representation of consecutive patients, including all individuals regardless of their entry date into the study is the methodologically correct and standard approach for time-to-event analyses. | **Added to the manuscript Page 5 Line 6-7:** Patients who were alive at the time of the last follow-up or were lost to follow-up were right-censored at the date of their last clinical examination. |
| Details of patient symptomatology and imaging strategy are not mentioned. This is also important as many patients with end stage tumors carry asymptomatic "silent" CM. If only symptomatic patients were referred, then many patients with peripheral or small asymptomatic lesions are missed from the sample. | We thank the reviewer for this comment and agree that many end stage cancer patients likely carry “silent” choroidal metastases that go clinically undetected. To address this important point, we have now added the patient symptomatology (Symptomatic at presentation, n (%)) to the revised manuscript and provided the exact patient numbers in Table 2.  Previously noted in the literature, the true prevalence of choroidal metastasis is undoubtedly higher than observed in clinical settings^8^. *(Eliassi-Rad, B., Albert, D. M. & Green, W. R. Frequency of ocular metastases in patients dying of cancer in eye bank populations. Br. J. Ophthalmol. 80, 125–128 (1996)).* Many palliative patients are in a poor general condition, making them less likely to notice peripheral visual changes or to attend routine ophthalmic examinations. Furthermore, within the Austrian healthcare system, current general oncological guidelines do not mandate routine ophthalmic screening for metastatic spread to the choroid in the absence of ocular suspicion which also can be found in the literature^4^ *(Kreusel, K. M. et al. Choroidal metastasis in disseminated lung cancer: frequency and risk factors. Am. J. Ophthalmol. 134, 445–447 (2002).* Consequently, our cohort naturally includes a predominantly symptomatic patient population who were referred specifically due to visual complaints.  Regarding a specific imaging strategy for general screening, we believe that evaluating systemic screening protocols falls outside the primary scope of this manuscript, which is strictly focused on survival predictors once a choroidal metastasis has been diagnosed. | Sympomatic at presentation added to **Table 2** including numbers. |
| Treatment details are not incorporated in the analysis. Patients who elect for best supportive care would not have similar survival rates to those who elect for palliative radiation for example. | Again, this is an interesting question but this is beyond the scope of our research question. We focused on appearance of metastatic spread to the choroid.  Due to the retrospective character and the collected data from different hospitals from all over Austria, this data was impossible to collect. |  |
|  |  |  |
| Minor Comments |  | Change in the Manuscript |
| Introduction too lengthy, unnecessary to convey the background and gap of knowledge. Irrelevant sections on incidence data of primary tumors, orbital mets, screening methods, and treatment modalities - none of which are the focus of the manuscript. | We shortened the introduction to better focus on the important topics. | **Deleted from the manuscript Page2 Line 2-8:** "The incidence of breast cancer in Europe is approximately 530,000 new cases per year, making it the most common cancer among women, while lung cancer accounts for about 480,000 new cases annually and is the leading cause of cancer death overall. In the last two decades, breast cancer mortality has fallen by 30% due to screening, earlier diagnosis and more effective systemic therapies including endocrine treatment, chemotherapy, and HER2-targeted agents 5.6. Lung cancer outcomes have improved more modestly, especially in non-small cell lung cancer, driven by molecular diagnostics, targeted therapy and immunotherapy as well as earlier detection7-9."  **Deleted Page 2 Line 29-35: “**Screening of uveal metastases involves various imaging modalities, including B-scan ultrasonography, fluorescein angiography, optical coherence tomography (OCT) and ultra-widefield fundus photography 30–33. Treatment strategies for intraocular metastases depend on the patient’s general condition, the status of the primary tumor, as well as the location and the number of the metastatic lesions 34–37. Treatment is often palliative. External beam radiotherapy is commonly used for symptomatic lesions, often achieving local control and preservation of vision38,39.) |
| Introduction focuses on data from Europe and not globally (readership of the audience). | Due to the comment above: on incidence of primary tumors. This section was deleted, shortened and does not focus on European data anymore. | **Deleted from the manuscript Page2 Line 2-8:** "The incidence of breast cancer in Europe is approximately 530,000 new cases per year, making it the most common cancer among women, while lung cancer accounts for about 480,000 new cases annually and is the leading cause of cancer death overall. In the last two decades, breast cancer mortality has fallen by 30% due to screening, earlier diagnosis and more effective systemic therapies including endocrine treatment, chemotherapy, and HER2-targeted agents 5.6. Lung cancer outcomes have improved more modestly, especially in non-small cell lung cancer, driven by molecular diagnostics, targeted therapy and immunotherapy as well as earlier detection7-9." |
| Methods: Unclear why patients with bilateral involvement only had 1 eye included (right eye). Bilateral involvement should have been incorporated as a variable for survival analysis. Also, the worse eye (larger lesion) should have been included in dimension measurements. | We thank the reviewer for these important methodological questions. The decision regarding the handling of bilateral cases was strictly driven by statistical limitations and data availability to ensure the robustness of our survival model. However, we have included the numbers in the manuscript already: see Table 2 in the manuscript: unilateral ocular manifestation.  First, regarding the inclusion of "bilateral involvement" as a separate variable: Given our overall cohort size of 70 patients, the number of outcome events (deaths) limits the number of independent variables we can reliably include in a multivariate Cox regression model. Adding another predictor to the multivariate analysis would have led to an overfitted model, resulting in a severe loss of statistical power and compromising the reliability of the entire analysis.  Second, regarding the inclusion of only one eye: Including both eyes of the same patient in the survival analysis would violate the fundamental statistical assumption of independent observations. Therefore, analyzing a single eye per patient is mandatory. While selecting the "worse eye" (largest lesion) is a valid alternative approach, this was unfortunately not feasible in our cohort due to the missing ultrasound dimension data in some cases. Relying on lesion size to select the study eye would have forced us to exclude these patients entirely.  Therefore, we opted for a standardized, predefined selection (specifically, the right eye) to maintain strict objectivity, prevent any potential selection bias regarding tumor dimensions, and maximize overall patient inclusion. |  |
| Methods: imaging protocols should have been mentioned, especially since the authors report rates of imaging signs (e.g., SRF), it is thus important to mention what type of OCT protocol was used for detection of the fluid. | Fluid was added to the analysis as a dichotomous variable (yes/no); therefore, the type of OCT and imaging protocols are of no specific interest in this study. |  |
| Methods: US is very operator-dependent and the authors should mention if measurements were done by a single operator to avoid bias. | Intraoperator differences are beyond the scope of this manuscript. US is based on a standardized protocol, comprising longitudinal and transversal US through the apex of the lesion performed by trained ocular oncologists. The protocol is mentioned in the methods section: page 4, line 30-38. |  |
| Statements like "A total of 70 patients... (table 1)" belong to the Results and not Methods section. | The statement was changed. | **Changed Page 5 Line 3-4**: Choroidal metastases originating from 14 subgroups of primary tumors were analyzed. |
| Results: missing data on US dimensions limit the ability to include volumetric data as a variable in the analysis. | We thank the reviewer for this valid observation and agree that complete volumetric data would have been a valuable addition.  However, we opted against including these parameters in our multivariate analysis for both technical and statistical reasons.  First, despite exhaustive efforts to retrieve this information from all current and historical databases, legacy devices, and medical records dating back to 2013, exact three-dimensional measurements were sometimes unavailable. Specifically, in the earlier years of our inclusion period, standard clinical documentation primarily focused on recording only the tumor height rather than full volumetric parameters.  Therefore, the missing volumetric data does not result from a lack of retrieval efforts, but rather reflects the evolving clinical documentation standards to long-term retrospective studies.  The volumetric approach used is a standard procedure in ocular oncology, as it has been abundantly used in choroidal melanoma. It is evident that this approach does not allow for exact volumetric assessments, but allows to compare groups. A reason why we did not include this parameter in our model.  Second, our preliminary univariate analysis showed no significant association between volumetric tumor dimensions and survival. Consequently, as these parameters did not demonstrate prognostic relevance in our cohort, they were excluded from the final multivariate model. Given our sample size of 70 patients, including additional non-significant variables would have unnecessarily consumed degrees of freedom, reducing the statistical power of the model and increasing the risk of overfitting. | **Added to the Manuscript Page 9 Line 34-36:** Moreover, exact volumetric ultrasound data could not be included in the analysis, as historical clinical documentation primarily focused on tumor height rather than full three-dimensional measurements. |
| Results: very wide CI in Cox analysis denoting heterogeneity. | We appreciate the reviewer’s comment regarding the confidence intervals (CIs). While we agree that the CIs reflect the natural clinical variance of our cohort, we feel they are actually quite reasonable given the reality of studying a rare condition like choroidal metastasis.  Specifically, the 95% CIs for age (1.15 - 3.82) and the number of extra-ocular metastases (1.10 - 5.83) are well within the expected statistical range for an ophthalmic oncology cohort of 70 patients. As is common in rare disease research, grouping patients for analysis naturally creates smaller subgroups - such as the 13 patients with ≤1 non-ocular metastasis. In a Cox regression model, this limited number of cases and events broadens the intervals.  Nevertheless, both predictors remained robustly significant (p = 0.016 and p = 0.029, respectively). We chose to retain these variables because, from a clinical perspective, they are important indicators of overall disease burden when counseling patients. We have ensured that the limitations section clearly addresses these small subgroup sizes, allowing readers to interpret the hazard ratios in their proper clinical context. | **Added to the Manuscript Page 9 Line 27- 30:** Furthermore, the overall limited sample size and the subsequent formation of even smaller subgroups for categorical variables (e.g., n = 13 for patients with ≤1 non-ocular metastasis) mathematically resulted in wide confidence intervals in our multivariate analysis; therefore, these specific hazard ratios should be interpreted with appropriate clinical caution |
| Discussion: too lengthy. | We thank the reviewer for the suggestion. We have shortened the Discussion to improve clarity which you can see in the revised manuscript with track changes. Additionally, we expanded the Limitations section to ensure greater transparency regarding our methodology. | **Page 8:** Line 20-29, Line 32-37 restructured and shortened, **Page 9:** Line 4-12 deleted, Line 18-26 deleted, Line 28-30 deleted, **Page 10**: line 4-6 deleted, Line 11-14 deleted, Line 15-24 deleted, line 36-38 deleted.  Added to the manuscript: **Page 10** Line 38- **Page** **11** Line 9, Line 13-26. |
