# Supplementary material for: Choroidal metastasis: Impact of primary tumors and age on survival - a single center analysis
Source: PLoS One. 2026 Jul 27;21(7):e0354598. doi: 10.1371/journal.pone.0354598 (PMC13405101; doi:10.1371/journal.pone.0354598)
Supplement: Peer review history

# Author comment: Author response to Decision Letter 0

**DOI:** 10.1371/journal.pone.0354598.r001

**Transfer Alert**

This paper was transferred from another journal. As a result, its full editorial history (including decision letters, peer reviews and author responses) may not be present.

23 Apr 2026

# Peer review: Decision Letter 0

**DOI:** 10.1371/journal.pone.0354598.r002

**Authors:** Carmelo Caldarella

27 May 2026

PONE-D-26-15940

Choroidal Metastasis: Impact of Primary Tumors and Age on Survival - a single center analysis.

PLOS One

Dear Dr. Told,

Thank you for submitting your manuscript to PLOS ONE. After careful consideration, we feel that it has merit but does not fully meet PLOS ONE’s publication criteria as it currently stands. Therefore, we invite you to submit a revised version of the manuscript that addresses the points raised during the review process.

**ACADEMIC EDITOR:**

**Marin concerns:**  Better depiction of primary tumor histology; address heterogeneity in group three

Please submit your revised manuscript by Jul 11 2026 11:59PM. If you will need more time than this to complete your revisions, please reply to this message or contact the journal office at plosone@plos.org. When you're ready to submit your revision, log on to https://www.editorialmanager.com/pone/ and select the 'Submissions Needing Revision' folder to locate your manuscript file.

Please include the following items when submitting your revised manuscript:

- A letter that responds to each point raised by the academic editor and reviewer(s). You should upload this letter as a separate file labeled 'Response to Reviewers'.
- A marked-up copy of your manuscript that highlights changes made to the original version. You should upload this as a separate file labeled 'Revised Manuscript with Track Changes'.
- An unmarked version of your revised paper without tracked changes. You should upload this as a separate file labeled 'Manuscript'.

If you would like to make changes to your financial disclosure, please include your updated statement in your cover letter. Guidelines for resubmitting your figure files are available below the reviewer comments at the end of this letter.

If applicable, we recommend that you deposit your laboratory protocols in protocols.io to enhance the reproducibility of your results. Protocols.io assigns your protocol its own identifier (DOI) so that it can be cited independently in the future. For instructions see: https://journals.plos.org/plosone/s/submission-guidelines#loc-laboratory-protocols. Additionally, PLOS ONE offers an option for publishing peer-reviewed Lab Protocol articles, which describe protocols hosted on protocols.io. Read more information on sharing protocols at https://plos.org/protocols?utm_medium=editorial-email&utm_source=authorletters&utm_campaign=protocols.

As the corresponding author, your ORCID iD is verified in the submission system and will appear in the published article. PLOS supports the use of ORCID, and we encourage all coauthors to register for an ORCID iD and use it as well. Please encourage your coauthors to verify their ORCID iD within the submission system before final acceptance, as unverified ORCID iDs will not appear in the published article. *Only*  the individual author can complete the verification step; PLOS staff *cannot*  verify ORCID iDs on behalf of authors.

We look forward to receiving your revised manuscript.

Kind regards,

Carmelo Caldarella, Ph.D., M.D.

Academic Editor

PLOS One

Journal Requirements:

If the reviewer comments include a recommendation to cite specific previously published works, please review and evaluate these publications to determine whether they are relevant and should be cited. There is no requirement to cite these works unless the editor has indicated otherwise.

Additional Editor Comments:

Dear Authors, please revise your manuscript according to comments provided by the Reviewers, particularly addressing issues regarding the heterogeneity in group three and better depicting primary tumor histology

[Note: HTML markup is below. Please do not edit.]

Reviewers' comments:

Reviewer's Responses to Questions

**Comments to the Author**

1. Is the manuscript technically sound, and do the data support the conclusions?

The manuscript must describe a technically sound piece of scientific research with data that supports the conclusions. Experiments must have been conducted rigorously, with appropriate controls, replication, and sample sizes. The conclusions must be drawn appropriately based on the data presented.

Reviewer #1: Yes

Reviewer #2: Yes

Reviewer #3: No

**********

2. Has the statistical analysis been performed appropriately and rigorously?

Reviewer #1: Yes

Reviewer #2: I Don't Know

Reviewer #3: No

**********

3. Have the authors made all data underlying the findings in their manuscript fully available?

The PLOS Data policy requires authors to make all data underlying the findings described in their manuscript fully available without restriction, with rare exception (please refer to the Data Availability Statement in the manuscript PDF file). The data should be provided as part of the manuscript or its supporting information, or deposited to a public repository. For example, in addition to summary statistics, the data points behind means, medians and variance measures should be available. If there are restrictions on publicly sharing data—e.g. participant privacy or use of data from a third party—those must be specified.

Reviewer #1: Yes

Reviewer #2: Yes

Reviewer #3: No

**********

4. Is the manuscript presented in an intelligible fashion and written in standard English?

PLOS ONE does not copyedit accepted manuscripts, so the language in submitted articles must be clear, correct, and unambiguous. Any typographical or grammatical errors should be corrected at revision, so please note any specific errors here.

Reviewer #1: Yes

Reviewer #2: Yes

Reviewer #3: Yes

**********

5. Review Comments to the Author

Please use the space provided to explain your answers to the questions above. You may also include additional comments for the author, including concerns about dual publication, research ethics, or publication ethics. (Please upload your review as an attachment if it exceeds 20,000 characters)

Reviewer #1: The manuscript is very well written and a good point to make. Would accept as is, adds to the information already known for metastases and helps discuss prognosis with patients that come in with questions.

Reviewer #2: The uvea is the most common site of metastasis in the eye. For both ophthalmologist and general practitioners, this article provides highly valuable information that predicts the survival of patients with uveal mets resulting from primary tumours of the breast or lung and also relates the age of the patients at the time of uveal mets to the survival. The clinical implications of this paper are that we can better counsel our patients about their prognosis.

Reviewer #3: Fuchs and colleagues present results of a retrospective analysis of survival in patients with metastatic tumors to the choroid (CM). They found that older patient age and more than one choroidal lesion were predictors of mortality. The study is interesting in its concept as a counseling basis for patients with choroidal metastasis. However, several integral methodological flaws limits the ability to draw any solid conclusions.

Major comments:

- The major concern in the study is the lack of subtyping of the primary tumors, which is a major confounder for survival. For example, breast cancer is not a single disease but a heterogenous group of diseases. Triple negative disease has poor survival than ER/PR tumors or HER-2 enriched. Similarly, lung cancer has many subtypes with different survival patterns. The lack of incorporation of molecular subtypes introduces great bias in the results.

- The other major confounder not accounted for is the sites of extra-ocular metastasis; for example, metastatic disease to the brain carries much worse prognosis than to the bones.

- As the authors acknowledge in the limitations, group 3 is heterogenous ("Other"); each of the 12 malignancy types would carry different prognosis and grouping all types under one category is inappropriate. This third group should have been omitted.

- There was no minimum limit on follow-up duration mentioned by the authors. In that sense, patients with recent CM diagnosis were included and compared to patients with long term diagnosis, which would skew survival data.

- Details of patient symptomatology and imaging strategy are not mentioned. This is also important as many patients with end stage tumors carry asymptomatic "silent" CM. If only symptomatic patients were referred, then many patients with peripheral or small asymptomatic lesions are missed from the sample.

- Treatment details are not incorporated in the analysis. Patients who elect for best supportive care would not have similar survival rates to those who elect for palliative radiation for example.

Minor comments:

- Introduction too lengthy, unnecessary to convey the background and gap of knowledge. Irrelevant sections on incidence data of primary tumors, orbital mets, screening methods, and treatment modalities - none of which are the focus of the manuscript.

- Introduction focuses on data from Europe and not globally (readership of the audience).

- Methods: Unclear why patients with bilateral involvement only had 1 eye included (right eye). Bilateral involvement should have been incorporated as a variable for survival analysis. Also, the worse eye (larger lesion) should have been included in dimension measurements.

- Methods: imaging protocols should have been mentioned, especially since the authors report rates of imaging signs (e.g., SRF), it is thus important to mention what type of OCT protocol was used for detection of the fluid.

- Methods: US is very operator-dependent and the authors should mention if measurements were done by a single operator to avoid bias.

- Statements like "A total of 70 patients... (table 1)" belong to the Results and not Methods section.

- Results: missing data on US dimensions limit the ability to include volumetric data as a variable in the analysis.

- Results: very wide CI in Cox analysis denoting heterogeneity.

- Discussion: too lengthy.

**********

6. PLOS authors have the option to publish the peer review history of their article (what does this mean?). If published, this will include your full peer review and any attached files.

If you choose “no”, your identity will remain anonymous but your review may still be made public.

**Do you want your identity to be public for this peer review?**  For information about this choice, including consent withdrawal, please see our Privacy Policy.

Reviewer #1: No

Reviewer #2: No

Reviewer #3: No

**********

[NOTE: If reviewer comments were submitted as an attachment file, they will be attached to this email and accessible via the submission site. Please log into your account, locate the manuscript record, and check for the action link "View Attachments". If this link does not appear, there are no attachment files.]

To ensure your figures meet our technical requirements, please review our figure guidelines: https://journals.plos.org/plosone/s/figures

You may also use PLOS’s free figure tool, NAAS, to help you prepare publication quality figures: https://journals.plos.org/plosone/s/figures#loc-tools-for-figure-preparation.

NAAS will assess whether your figures meet our technical requirements by comparing each figure against our figure specifications.

# Author comment: Author response to Decision Letter 1

**DOI:** 10.1371/journal.pone.0354598.r003

11 Jun 2026

Thank you for the opportunity to submit our revised manuscript. We have addressed all editor and reviewer comments in detail. A comprehensive, point-by-point response to all queries, along with the manuscript and the corresponding tracking of changes in the text, has been provided in our uploaded 'Manuscript', 'Response to Reviewers' and "Revised Manuscript with Track Changes" file.

Attachment Submitted filename: Response to Reviewers.docx

# Peer review: Decision Letter 1

**DOI:** 10.1371/journal.pone.0354598.r004

**Authors:** Carmelo Caldarella

9 Jul 2026

Choroidal Metastasis: Impact of Primary Tumors and Age on Survival - a single center analysis.

PONE-D-26-15940R1

Dear Dr. Told,

We’re pleased to inform you that your manuscript has been judged scientifically suitable for publication and will be formally accepted for publication once it meets all outstanding technical requirements.

Within one week, you’ll receive an e-mail detailing the required amendments. When these have been addressed, you’ll receive a formal acceptance letter and your manuscript will be scheduled for publication.

An invoice will be generated when your article is formally accepted. Please note, if your institution has a publishing partnership with PLOS and your article meets the relevant criteria, all or part of your publication costs will be covered. Please make sure your user information is up-to-date by logging into Editorial Manager at Editorial Manager® and clicking the ‘Update My Information' link at the top of the page. For questions related to billing, please contact billing support.

If your institution or institutions have a press office, please notify them about your upcoming paper to help maximize its impact. If they’ll be preparing press materials, please inform our press team as soon as possible -- no later than 48 hours after receiving the formal acceptance. Your manuscript will remain under strict press embargo until 2 pm Eastern Time on the date of publication. For more information, please contact onepress@plos.org.

Kind regards,

Carmelo Caldarella, Ph.D., M.D.

Academic Editor

PLOS One

Additional Editor Comments (optional):

Reviewers' comments:

Reviewer's Responses to Questions

**Comments to the Author**

1. If the authors have adequately addressed your comments raised in a previous round of review and you feel that this manuscript is now acceptable for publication, you may indicate that here to bypass the “Comments to the Author” section, enter your conflict of interest statement in the “Confidential to Editor” section, and submit your "Accept" recommendation.

Reviewer #1: All comments have been addressed

**********

2. Is the manuscript technically sound, and do the data support the conclusions?

The manuscript must describe a technically sound piece of scientific research with data that supports the conclusions. Experiments must have been conducted rigorously, with appropriate controls, replication, and sample sizes. The conclusions must be drawn appropriately based on the data presented.

Reviewer #1: Yes

**********

3. Has the statistical analysis been performed appropriately and rigorously?

Reviewer #1: Yes

**********

4. Have the authors made all data underlying the findings in their manuscript fully available?

The PLOS Data policy requires authors to make all data underlying the findings described in their manuscript fully available without restriction, with rare exception (please refer to the Data Availability Statement in the manuscript PDF file). The data should be provided as part of the manuscript or its supporting information, or deposited to a public repository. For example, in addition to summary statistics, the data points behind means, medians and variance measures should be available. If there are restrictions on publicly sharing data—e.g. participant privacy or use of data from a third party—those must be specified.

Reviewer #1: Yes

**********

5. Is the manuscript presented in an intelligible fashion and written in standard English?

PLOS ONE does not copyedit accepted manuscripts, so the language in submitted articles must be clear, correct, and unambiguous. Any typographical or grammatical errors should be corrected at revision, so please note any specific errors here.

Reviewer #1: Yes

**********

6. Review Comments to the Author

Please use the space provided to explain your answers to the questions above. You may also include additional comments for the author, including concerns about dual publication, research ethics, or publication ethics. (Please upload your review as an attachment if it exceeds 20,000 characters)

Reviewer #1: Very well written and good responses and changes to the comments mentioned before. Would recommend accepting as is

**********

7. PLOS authors have the option to publish the peer review history of their article (what does this mean?). If published, this will include your full peer review and any attached files.

If you choose “no”, your identity will remain anonymous but your review may still be made public.

**Do you want your identity to be public for this peer review?**  For information about this choice, including consent withdrawal, please see our Privacy Policy.

Reviewer #1: No

**********

# Editor report: Acceptance letter

**DOI:** 10.1371/journal.pone.0354598.r005

**Authors:** Carmelo Caldarella

PONE-D-26-15940R1

PLOS One

Dear Dr. Told,

I'm pleased to inform you that your manuscript has been deemed suitable for publication in PLOS One. Congratulations! Your manuscript is now being handed over to our production team.

At this stage, our production department will prepare your paper for publication. This includes ensuring the following:

* All references, tables, and figures are properly cited

* All relevant supporting information is included in the manuscript submission,

* There are no issues that prevent the paper from being properly typeset

You will receive further instructions from the production team, including instructions on how to review your proof when it is ready. Please keep in mind that we are working through a large volume of accepted articles, so please give us a few days to review your paper and let you know the next and final steps.

Lastly, if your institution or institutions have a press office, please let them know about your upcoming paper now to help maximize its impact. If they'll be preparing press materials, please inform our press team within the next 48 hours. Your manuscript will remain under strict press embargo until 2 pm Eastern Time on the date of publication. For more information, please contact onepress@plos.org.

You will receive an invoice from PLOS for your publication fee after your manuscript has reached the completed accept phase. If you receive an email requesting payment before acceptance or for any other service, this may be a phishing scheme. Learn how to identify phishing emails and protect your accounts at https://explore.plos.org/phishing.

If we can help with anything else, please email us at customercare@plos.org.

Thank you for submitting your work to PLOS ONE and supporting open access.

Kind regards,

PLOS ONE Editorial Office Staff

on behalf of

Dr. Carmelo Caldarella

Academic Editor

PLOS One
